# Teaching appropriate interactions with pharmaceutical company representatives: The impact of an innovative workshop on student attitudes

**DOI:** 10.1186/1472-6920-5-5

**Published:** 2005-02-08

**Authors:** James L Wofford, Christopher A Ohl

**Affiliations:** 1 Department of Internal Medicine, Wake Forest University School of Medicine, Winston-Salem, NC, USA

## Abstract

**Background:**

Pharmaceutical company representatives (PCRs) influence the prescribing habits and professional behaviour of physicians. However, the skills for interacting with PCRs are not taught in the traditional medical school curriculum. We examined whether an innovative, mandatory workshop for third year medical students had immediate effects on knowledge and attitudes regarding interactions with PCRs.

**Methods:**

Surveys issued before and after the workshop intervention solicited opinions (five point Likert scales) from third year students (n = 75) about the degree of bias in PCR information, the influence of PCRs on prescribing habits, the acceptability of specific gifts, and the educational value of PCR information for both practicing physicians and students. Two faculty members and one PCR led the workshop, which highlighted typical physician-PCR interactions, the use of samples and gifts, the validity and legal boundaries of PCR information, and associated ethical issues. Role plays with the PCR demonstrated appropriate and inappropriate strategies for interacting with PCRs.

**Results:**

The majority of third year students (56%, 42/75) had experienced more than three personal conversations with a PCR about a drug product since starting medical school. Five percent (4/75) claimed no previous personal experience with PCRs. Most students (57.3%, 43/75) were not aware of available guidelines regarding PCR interactions. Twenty-eight percent of students (21/75) thought that none of the named activities/gifts (lunch access, free stethoscope, textbooks, educational CD-ROMS, sporting events) should be restricted, while 24.0% (8/75) thought that students should be restricted only from sporting events. The perceived educational value of PCR information to both practicing physicians and students increased after the workshop intervention from 17.7% to 43.2% (chi square, p = .0001), and 22.1% to 40.5% (p = .0007), respectively. Student perceptions of the degree of bias of PCR information decreased from 84.1% to 72.9% (p = .065), but the perceived degree of influence on prescribing increased (44.2% to 62.1% (p = .02)).

**Conclusions:**

Students have exposure to PCRs early in their medical training. A single workshop intervention may influence student attitudes toward interactions with PCRs. Students were more likely to acknowledge the educational value of PCR interactions and their impact on prescribing after the workshop intervention.

## Background

Pharmaceutical company representatives (PCRs) influence the prescribing habits and professional behavior of physicians [1]. Despite the availability of guidelines regarding appropriate interactions with PCRs for practicing physicians [2-4], the skills for interacting with PCRs have not been included as part of the traditional medical school curriculum.

Physicians in training may be particularly susceptible to marketing strategies from PCRs. Restricting interactions between physician in training and PCRs is one approach to eliminating adverse effects of contacts with PCRs [5]. However, physicians in training will likely deal with such marketing influences once in practice. The provision of training or guided experiences in dealing with PCRs seems a more reasonable educational strategy for producing a physician who will be aware of the potential conflict of interest from the profit motive inherent in the pharmaceutical and other health related industries. To our knowledge, there is only one published study of an educational intervention targeting third year medical students on the subject of appropriate interactions with PCRs [6]. Furthermore, the best means of developing the skills and attitudes for interacting appropriately with PCRs is not well defined.

We sought to examine whether a single workshop intervention had immediate effects on the attitudes of third year medical students regarding interactions with PCRs. The goal of the workshop was to increase the student's knowledge and awareness of ethical issues surrounding the PCR encounter, and to improve the students' interactions with representatives by fostering discussion on the profit motive in pharmaceutical marketing, PCR marketing techniques, and appropriate interactions with the PCR, issues necessary for critical thinking about the potential conflict of interest. Unique to our intervention was the participation of a PCR who role played a typical PCR encounter and who offered a perspective on marketing from the perspective of industry. Our findings have implications for institutions considering strategies for controlling PCR interactions and for medical educators seeking to develop curricula for marketing in medicine.

## Methods

During the ambulatory internal medicine clerkship of the third year medical school curriculum, students were required to attend a ninety minute workshop entitled "Appropriate Encounters with Pharmaceutical Representatives". These workshops took place three times during the calendar year 2001 for three different student groups. To the best of our knowledge, there were no other organized learning experiences in the medical school curriculum about interactions with pharmaceutical representatives, either before or during the study period. We did not seek approval by the institutional review board for ethical research practice at our institution, because at that time the study was conducted, approval of student education projects was considered unnecessary.

Two faculty members interested in the subject (JW, CO), and a regional manager of pharmaceutical representatives from a major pharmaceutical company facilitated the ninety minute workshop. The workshop began by soliciting student opinions regarding the characteristics of typical interactions with PCRs. After a list of characteristics was compiled, each characteristic was discussed in more detail and compared with previous personal experiences with PCRs. Salient points of the subsequent discussion included the usefulness of patient assistance programs, the use of samples and gifts in PCR marketing strategies, the validity and legal boundaries of information provided by the PCR, and the ethical and legal aspects of physician-industry relations. The final segment of the workshop involved two student volunteers who role played a typical PCR encounter in the office. After discussion of the first role play, a second role play between one faculty member (CO) and the PCR demonstrated desirable characteristics of the PCR encounter.

A pre-intervention survey handed out and collected prior to the beginning of the workshop solicited information about the number of previous personal experiences with PCRs, and whether the student was previously aware of guidelines (medical school, federal government, or professional society) for appropriate interactions with PCRs. Using five point Likert scales, the survey solicited student attitudes about the educational value of PCR information for practicing physicians and for medical students, the degree of bias in PCR information, and the degree of influence of PCRs on prescribing habits. One additional question solicited the acceptability to students of specific gifts (lunch access, free stethoscope, textbooks, educational CD-ROMS, sporting events) from PCRs. A post-intervention survey with the same attitude questions was administered and collected as students left the workshop. The available data comes from three groups – the third, or last student group of academic year 2000–1 and the first two student groups of academic year 2001–2.

Students were characterized by gender, age, and number of previous personal contacts with PCRs (None, 1–3, 4–6, >6). For the purposes of understanding the Likert scale responses for student attitudes, we collapsed Likert scores into three categories – scale responses of 1 or 2 to signify disagreement, a scale response of 3 to signify neutral, and a response of 4 or 5 to signify agreement with the attitude question. We compared student attitudes toward the educational value of PCR detailing for medical students with the perceived value for practicing physicians using the Pearson chi square test.

The association between previous personal PCR experience and attitudes about the educational value of PCR detailing was explored using analysis of variance. We also compared attitudes before and after the workshop intervention using the Pearson chi square test and a dichotomous variable a response of 4 or 5 (versus 3 or lower) on the Likert scale.

## Results

### Student characteristics

A total of 75 students attended one of the three mandatory workshops on "Appropriate Encounters with PCRs". One student did not complete a post-intervention survey. The mean age of students was 26.4 (± 2.4), and males (56.1%, n = 41) outnumbered females. Fifty-six percent of students (42/75) had experienced more than three personal conversations about a pharmaceutical product with a PCR since starting medical school. Five percent (4/75) claimed no previous personal experience with PCRs. There was no association between the number of PCR contacts, and either gender, age or time of the academic year.

### Student attitudes toward value of PCR interaction

The pre-intervention survey showed that PCR detailing was of educational value to 22.1% (18/75) of students with no perceived difference in educational value to medical students versus that to practicing physicians (chi square, p = .40). While students agreed that the degree of bias from PCR information was substantial (86.7%, 65/75), only 44.0% (33/75) of students felt that pharmaceutical representatives were influential with regard to physicians' prescribing habits. No relationship between number of previous personal PCR contacts and educational value to medical students was demonstrated (ANOVA p = .08)

### Awareness of guidelines and attitudes toward gifts

Forty-three percent (32\75) of students reported awareness of available guidelines regarding PCR interactions. Fifty percent (16/32) of those students reported familiarity with medical school guidelines, 21.8% (7/32) with federal government guidelines, and 40.6% (13/32) with professional society guidelines. When asked which drug company sponsored activities/gifts targeting medical students should be restricted, 28.0% of students (21\75) thought that none of the named activities/gifts (lunch access, free stethoscope, textbooks, educational CD-ROMS, sporting events) should be restricted. Twenty-four percent (8/75) of students thought that only sporting events should be restricted.

### Effect of workshop intervention on student attitudes

**Figure 1:**
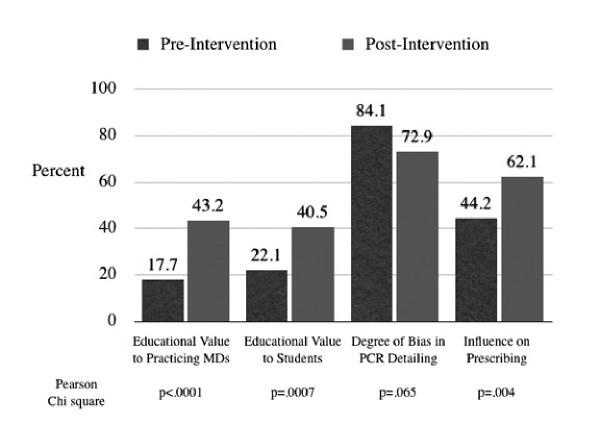
**Student attitudes toward PCR detailing before and after workshop intervention**. Student perception of the educational value of PCR interactions increased after the intervention at the same time that the perception that PCRs influenced prescribing increased. Student perception of the degree of bias decreased slightly after the workshop, but this decrease was not statistically significant.

Figure 1 shows that the perceived educational value to both practicing and student physicians increased after the workshop intervention from 17.7% to 43.2% (chi square, p = .0001), and 22.1% to 40.5% (p = .0007), respectively. Fifty-eight percent of students (43/74) and 37.8% (28/74), respectively, changed their Likert scale response to the questions on educational value by at least one point after the workshop intervention (Table 1). Student perceptions of the degree of bias of PCR information decreased from 84.1% to 72.9% (p = .065), but the perceived degree of influence on prescribing increased (44.2% to 62.1% (p = .02)). The response to the question on the degree of bias in PCR detailing changed by at least one Likert scale point for 17 students (17/74, 23.0%) (Table 1). The response to the question on the influence of PCR detailing on prescribing practices changed by at least one Likert scale point for 34 students (34/75, 46.0%).

**Table 1:** Student attitudes toward PCR detailing before and after workshop intervention

In your opinion, what is the educational value to practicing physicians offered by detailing from pharmaceutical representatives? (1 = No value at all, 5 = Extremely valuable)				
	Before-Not Valuable (<3)	Before-Neutral (3)	Before-Valuable (>3)	

After-Not valuable (<3)	8	1	0	9
After-Neutral (3)	10	20	3	33
After-Valuable(>3)	2	18	12	32
	20	39	15	

In your opinion, what is the educational value to medical students offered by detailing from pharmaceutical representatives? (1 = No value at all, 5 = Extremely valuable)				

	Before-Not Valuable (<3)	Before-Neutral (3)	Before-Valuable (>3)	

After-Not valuable (<3)	13	0	0	13
After-Neutral (3)	8	19	4	31
After-Valuable(>3)	6	10	14	30
	27	29	18	

What is you perception of the degree of bias in the information provided by pharmaceutical representatives detailing to practicing physicians? (1 = Not at all biased, 5 = Totally biased)				

	Before-Not Biased (<3)	Before-Neutral (3)	Before-Biased (>3)	

After-Not biased (<3)	0	1	1	2
After-Neutral (3)	0	6	12	18
After-Biased(>3)	0	3	51	54
	0	10	64	

How influential are pharmaceutical representatives with regard to physicians' prescribing habits? (1 = Not at all influential, 5 = Very influential)				

	Before-Not Influential (<3)	Before-Neutral (3)	Before-Influential (>3)	

**After-Not influential (<3)**	2	2	1	5
After-Neutral (3)	7	11	5	23
After- Influential (>3)	2	17	27	36
	11	30	33	

## Discussion

Professional relationships with PCRs begin early in a physician's career. Because physicians typically underestimate the influence of pharmaceutical marketing on prescribing practices, countering this naivete early is warranted. Because students do not yet have prescribing privileges, the effect of PCR detailing on the prescribing decision is less relevant than how PCR contact shapes professional values [1]. Several studies have examined perceptions of the potential influence of PCRs on resident and practicing physicians [7-13]. However, fewer studies have examined the views of medical students [6,14,15].

Our data show that students have early exposure to PCRs, perhaps earlier than previously suspected. Only five percent of third year students at this institution had not yet experienced PCR detailing, and many had experienced greater than six such encounters. Although resident physicians understandably have more PCR contacts, on average at least three PCR encounters per month [11,16], to our knowledge, there are no studies of comparable data for medical students.

In this study few students were aware of existing guidelines for PCR interactions. Furthermore, students perceived information from PCRs to have educational value, and a value equivalent to that for practicing physicians. That students felt no more or less susceptible to marketing influence than practicing physicians is a marked contrast to the opinions of many educators that physicians in training need special protection from marketing influences [1,2,17].

Several interventions have been proposed for educating physicians-in-training about pharmaceutical marketing practices. Shaughnessy et al described a single brief seminar of marketing concepts followed by regular structured evaluation of PCR sales presentations throughout the following year [18]. Despite this well organized effort, meaningful educational outcomes were meager for the twelve residents evaluated. Hopper et al showed that a single elective forty minute lecture/discussion on ethical and marketing issues in pharmaceutical promotion was successful in improving attitudes and knowledge among residents and faculty [19]. They presented six vignettes to illustrate marketing techniques related to gifts, guidelines, and the yield of marketing for pharmaceutical companies. Vinson et al showed that a fifty minute lecture for first and second year medical students could have immediate effects on knowledge as measured by repeat anonymous survey six weeks later [15]. Palmisano et al described a ninety minute lecture and role-play with simulated PCRs to teach analysis of advertising copy and sales techniques, although no data on educational outcomes were offered [20]. Most similar to our intervention was this study by Wilkes and Hoffman who used pharmacists who were trained to portray PCRs during a one hour seminar targeting third year medical students [6]. Designed to promote critical thinking about appropriate physician-PCR interactions, the single workshop was successful in increasing the amount of uncertainty students felt about the accuracy and ethics of standard drug "detailing".

Similar to these interventions, our workshop intervention targeting third year medical students was a one time intervention and brief in duration. Our intervention differed in that the workshop (1) took place during the clinical clerkship year, (2) made use of practicing PCRs and physicians, and (3) encouraged a distanced but amicable relationship with the PCR. In contrast to many educators who oppose PCR contact for trainees, we encouraged respect for the individual PCR and the success of the pharmaceutical company's business model. We were concerned enough about the growing influence of pharmaceutical marketing on trainees to stage this workshop, but we were careful to remember our goals of encouraging critical thinking about the topic rather than simply condemning the PCR contact. As an example, we emphasized the legal limits regarding what the PCR could and could not say to the physician. This may explain, in part, why attitudes toward PCR information improved after the intervention, at the same time that perceived degree of influence on prescribing increased. Teaching students the "rules of the game" in PCR encounters may explain why students thought that PCR information had more educational value after the workshop.

The limitations of this study should be recognized. First, our survey took place at a single academic medical center affiliated with a private hospital. Student attitudes might be different at a state supported institution or at institutions where there are restrictions on the activities of PCRs. Second, students may have answered the post-intervention survey in a socially desirable manner. Respect for the PCR and the business model was presented as a balanced perspective toward marketing in medicine. Third, how immediate changes in student perceptions will ultimately translate into durable attitude changes and prescribing practices was not a goal of this study. We are not naïve enough to be certain that a one time intervention on any subject matter related to ethical issues will change student attitudes in a way that is durable. However, our goal as educators should be to make the students think critically, and demonstrating self-reported attitude changes is a necessary first step toward more durable change.

Some academic institutions have chosen to ban PCRs from the academic learning environment. McCormick et al showed that restricting access to PCRs during residency training was associated with less informational dependence on the PCR and a decreased frequency of PCR contact after training [5]. Underlying such a restrictive policy is the idea that trainees are not able and/or educable to resist the marketing tactics of PCRs. In contrast, our approach is based on the opinion that learning the skills for interacting appropriately with PCRs should not be delayed until a physician has entered practice, and that banning PCRs may simply extend the period of naivete for physicians in training [14]. Not only does such an omission in the curriculum miss the opportunity to teach physicians about professional relationships surrounding the business model. It also ignores the cost containment needs of the academic medical center and prospective employers.

The challenge for medical educators is how to incorporate this increasingly important knowledge domain into training programs. What aspects of marketing strategy need to be taught and how? The growing emphasis on social justice and professionalism should encourage the appropriate distance from and respect for marketing pressures in medicine and add support for this curricular element in medical education [21]. Our model suggests the possibility of a partnership between the pharmaceutical industry and educators in better preparing phsyicians in training for marketing in medicine. While pharmaceutical companies are the current target of criticism of commercialism in medicine, other services (durable medical equipment, herbal/nutritional supplements, new medical technologies) are all accompanied by marketing pressures that physicians will have to factor into clinical decision making.

## Conclusions

Medical students have exposure to PCRs early in their medical education, at least in this setting. Students perceived information from PCRs to have moderate educational value, and a value equivalent to that for practicing physicians. A brief workshop intervention can have a measurable immediate effect on student attitudes.

## List of abbreviations

PCR – pharmaceutical company representative

## Competing interests

The author(s) declare that they have no competing interests.

## Authors' contributions

JW conceived of the study, participated in its design and coordination, and drafted the manuscript.

CO participated in design and coordination of the study, conducted the intervention, participated in data interpretation, and manuscript revision.

Both authors read and approved the final manuscript.

## Pre-publication history

The pre-publication history for this paper can be accessed here:


